# Combined sprint and resistance training abrogates age differences in somatotropic hormones

**DOI:** 10.1371/journal.pone.0183184

**Published:** 2017-08-11

**Authors:** Maha Sellami, Wissem Dhahbi, Lawrence D. Hayes, Johnny Padulo, Fatma Rhibi, Hanen Djemail, Anis Chaouachi

**Affiliations:** 1 Tunisian Research Laboratory “Sport Performance Optimization” National Center of Medicine and Science in Sports, Tunis, Tunisia; 2 Active Ageing Research Group, Department of Medical and Sport Sciences, University of Cumbria, Bowerham Road, Lancaster, United Kingdom; 3 University eCampus, Novedrate, Italy; 4 Faculty of Kinesiology, University of Split, Split, Croatia; 5 Movement, Sport, Health and Sciences Laboratory (M2S), University of Rennes 2, Rennes, France; 6 Military Hospital of Instruction of Tunis, Department of Endocrinology, Tunis, Tunisia; Universidad Europea de Madrid, SPAIN

## Abstract

The aim of this investigation was to compare serum growth hormone (GH), insulin-like growth factor-1 (IGF-1) and insulin-like growth factor-binding protein-3 (IGFBP-3) in response to a combined sprint and resistance training (CSRT) program in young and middle-aged men.Thirty-eight healthy, moderately trained men participated in this study. Young and middle-aged men were randomly assigned to, a young training group (YT = 10, 21.4±1.2yrs) ora young control group (YC = 9, 21.6±1.8 yrs), a middle-aged training group (MAT = 10, 40.4±2.1 yrs) or a middle-aged control group (MAC = 9, 40.5±1.8 yrs). Participants performed the Wingate Anaerobic Test (WAnT) before and after a 13-week CSRT program (three sessions per week). Blood samples were collected at rest, after warm-up, immediately post-WAnT, and 10 min post-WAnT. CSRT induced increases in GH at rest and in response to the WAnT in YT and MAT (P<0.05). CSRT-induced increases were observed for IGF-1 and IGFBP-3 at rest in MAT only (P<0.05). Pre-training, GH, IGF-1 and IGFBP-3 were significantly higher at rest and in response to the WAnT in young participants as compared to their middle-aged counterparts (P<0.05). Post-training, YT and MAT had comparable basal GH (P>0.05). In response to the WAnT, amelioration of the age-effect was observed between YT and MAT for IGF-1 and IGF-1/IGFBP-3 ratio following CSRT (P>0.05). These data suggest that CSRT increases the activity of the GH/IGF-1 axis at rest and in response to the WAnT in young and middle-aged men. In addition, CSRT reduces the normal age-related decline of somatotropic hormones in middle-age men.

## Introduction

The aging process is associated with a precipitous decline in skeletal muscle mass and strength, estimated as 35–40% between 20 and 80 yrs[1], with an accelerated decline after 50 yrs[2]. Moreover, cross-sectional studies have observed that the ability to develop muscle strength and power declines from 40 to 80 yrs[3, 4]. It has been suggested that reduced muscle function may result from neural degeneration combined with muscle atrophy. Muscle atrophy occurs when muscle protein degradation exceeds muscle protein synthesis. Said muscle atrophy may be partly attributable to a reduction in anabolic hormone production [5], whilstage-associated physical inactivity may exacerbate muscle loss. Moreover, the reduction in systemic anabolic hormones may be further exacerbated by physical inactivity in older adults[6].

The systemic reduction in insulin-like growth factor-1 (IGF-1)has been attributed, in part, to decreased secretion of growth hormone (GH), the main secretagogue of IGF-1. GH secretion reduces ~14% per decade after the second decade [7], and reaches, by the age of ~60 yrs, half of the GH secretion of younger counterparts (20–30 yrs[8]). IGF-1, the main stimulated protein downstream of GH, concomitantly decreases with age (~10% per decade) and is associated with cell proliferation, cell differentiation, energy metabolism and prevention of apoptosis [9]. Most IGF-1 circulates in the blood bound to IGF-binding proteins (IGFBPs) which mediate bioavailability into tissue [10]. The most abundant is the insulin-like growth factor-binding protein 3 (IGFBP-3), which carries 90–95% of IGF-1 in circulation [11]. Hence, it is necessary to consider IGFBP-3 when total serum IGF-1 is determined to provide a measure of bioavailability. Serum IGFBP-3 is regulated by GH signaling [12, 13] and reduced with advanced age [14]. The main function of IGFBP-3 is to permit IGF-1 transport and to regulate anti-proliferative and apoptotic effects through cell surface receptor by opposing IGF-1 action [15, 16]. Moreover, IGFBP-3 is a valuable tool for diagnosis of GH perturbation during the aging process [17].

Because GH naturally declines with age, there has been increasing interest in the role of GH as an anti-aging factor, and some evidence suggests GH administration increases fatty acid oxidation, protein synthesis and, consequently, lean body mass [18]. To date, however, there have been no approved clinical studies to assess the effects of GH administration on muscle mass loss with aging. In fact, GH administration has been shown to have no effect on muscle function in healthy aged men and women [19].As GH administration is widely used as a muscle mass and performance enhancer [20], whether exercise could be a nonpharmacological intervention to enhance GH and subsequently muscle mass and muscle strength, requires further investigation.

Whilst acute exercise-induced elevations in GH and IGF-1 are consistently reported [20–26], the effect of long-term exercise training on basal GH and IGF-1 secretion is more ambiguous. It is well known that GH response exercise training depends on several factors including age, diet, stress, or training intensity [20, 27]. Metabolic (i.e glucose) and hormonal (i.e. catecholamines, cortisol, testosterone) factors also influence GH [28–30] which in turn are also dependent upon exercise intensity and training type[31, 32]. In this context, increase of these hormones is greater following anaerobic training in young and middle-aged trained men [6, 31–33]. Moreover, Nevill et al. [34] suggested that serum GH response to treadmill running was greater in sprinters compared to endurance athletes. In addition, endurance training (running for eight weeks) resulted in increased systemic IGF-1 (+15%) in subjects aged ~66 yrs [35], yet Vitiello, Wilkinson [36] observed no perturbation in IGF-1 amongst moderately and well-trained endurance athletes aged ~69 yrs. As such, intensive training using sprint or resistance training appears necessary to induce changes to serum GH. Moreover, numerous studies suggest sprint training may elicits greater increase in muscle strength than the endurance training[31, 34]. Borst et al. [37] suggested that 25 weeks’ resistance training in middle-aged men (~37 yrs) resulted in increased GH and IGF-1 with increase in strength performances. However, Adams et al. [38] suggested it necessary to add different types of exercise to resistance exercise in order to obtain higher muscle power during sprint exercise. In fact, some authors suggest combined sprint and resistance training is more efficient than sprint training only [39] or resistance training only [40].

Most existing GH and IGF-1 exercise studies have focusedon young (~20 yrs) and elderly (>65 yrs) subjects. Therefore, there is a paucity of data concerning the effect of concurrent exercise training (sprint interval and resistance exercises) on somatotropic hormones in adults ~40 yrs. Thus, we examined the effect of 13 weeks’concurrenttraining on GH, IGF-1, and IGFBP-3 in young (~20 yrs) and middle-aged (~40 yrs) men before and after supramaximal exercise. We hypothesized *a priori* that younger participants would have greater GH, IGF-1, and IGFBP-3. Moreover, we hypothesized that training would increase somatotropic hormone concentrations in both age groups.

## Materials and methods

### Participants

Military Participants reviewed and signed consent forms specifically approved by the “*Department of staff and training committee*” (Bouchoucha, Tunisia). The “*Departement of staff and training committee*" approved the entire study design which has been conducted according to the principles expressed in the Declaration of Helsinki.

During the design of the study, statistical power analysis was carried out to calculate sample size. This procedure showed that nine subjects for each of four groups were needed to achieve a statistical power of 80% to detect a small effect (*d* = 0.29) when assessed by four-factor mixed analysis of variance (ANOVA) with a level of significance of 5%. Therefore, thirty-eight healthy, moderately trained men (military participants) were recruited for participation in the present investigation.

To assess the physical condition of military participants, we used an adapted version of the Baeckequestionnaire [41]. Before the study, subjects performed 1 h of running and leisure-time physical activity at least three times per week (180 min·week^-1^). Inclusion criteria included the absence of the following: contraindications to maximal exercise testing (e.g., cardiovascular or pulmonary disease); endocrine disorders; metabolic syndrome symptoms (e.g., hypertension, impaired fasting glucose).Following recruitment and familiarization, participants completed medical history and dietary questionnaires. Thereafter, young and middle-aged participants were randomized to receive 13 weeks’ combined sprint and resistance training (CSRT), or control. Therefore, four groups existed: a young training group (YT = 10; age 21.4±1.2yrs, body height 178.3±3.2 cm, body mass 74.4±5.4 kg), a young control group (YC = 9; age 21.6±1.8 yrs, body height 179.7±6.4 cm, body mass 69.5±7.3 kg), a middle-aged training group (MAT = 10; age 40.4±2.1 yrs, body height 175.8±5.2 cm, body mass 78.4±5.2 kg) and a middle-aged control group (MAC = 9; 40.5±1.8 yrs, body height 177.3±4.4 cm, body mass 76.6±3.9 kg).

A conventional dietary survey was conducted by a sports nutritionist of the Department of Physical Education and Military Sport to monitor individual participants’ diets over the 13 weeks. Participants were asked to abstain from high glycemic loads, saturated and trans-fatty acids, caffeine, alcohol, drugs, vitamins or supplements, and low-fiber diets for the duration of the study.

Before training (during the medical examination), subjects were familiarized with testing procedures to negate learning effect. Participants avoided physical activity for 48 h preceding each test. The testing period was divided into two phases: before (P1), and after (P2), training. Each period lasted seven days and included anthropometric measurements andtwo consecutive laboratory visits separated by 48 h. The second phase (P2) began 48 h after training cessation and finished seven days later. All tests were performed in the morning 2h postprandial (standard breakfast: 10 kcal·kg^-1^, 55% carbohydrate, 33% lipids and 12% protein). Anthropometric parameters were measured on the morning of the first day. Measurement of body mass (kg) and height (cm) were taken from all participants. Body mass was measured to the nearest 0.1 kg, with subjects in light clothing and without shoes, using electronic scales (Kern, MFB 150K100). Height was determined to the nearest 0.5cm with a measuring tape fixed to the wall. Subsequently, skin-folds were measured using Harpenden calipers (Harpenden skinfold calipers, Sweden). Percentage body fat was determined by the four skin-folds method [42].

### Exercise tests

During the medical exam, participants performed the Astrand-Ryhming test [43]on a cycle ergometer (Monark Ergoline: ER900, Ergoline, Jaeger, Würzburg, Germany) to estimate maximal oxygen uptake (VO_2max_). Heart rate (S810, Polar Instruments Inc., Oulu, Finland) and rating of perceived exertion (RPE; Borg 1973) was recorded at the end of each stage. A maximal test was confirmed when participants achieved a minimum of any three of the following criteria; volitional exhaustion, peak heart rate within 10 beats of age predicted maximum, blood lactate above 8 mmol·L^-1^, final RPE>18 on Borg scale.

48 hours later (day 2), subjects performed a force-velocity (F/V) test [44] on a cycle ergometer (Monark Ergomedic 894E Peak Bike, Monark, Varberg, Sweden). The test began 5 min after warm-up (15 min at a power output corresponding to 50% estimated VO_2max_). This test comprised five short trials (6 s) against increasing resistance (2 kg each sprint) until the velocity began to decrease during the 6 s trials. Recovery time between each trial was 5 min. The highest pedaling cadence recorded after each trial was collected from a photoelectric cell fixed on the wheel of the cycle ergometer and connected to a computer. The load that permitted the highest peak power output was used for the Wingate Anaerobic Test (WAnT).

48 hours later (day 3), subjects performed the WAnT on a mechanically-braked Monark cycle ergometer (Monark Ergomedic 894E Peak Bike, Monark, Varberg, Sweden). The test commenced 5 min after warm-up. Subjects were asked to cycle maximally for 30s. Maximal power during the trial was considered as the highest value (W_peak_), while average power during the WAnT was considered as mean power (W_mean_).

Before the F/V test and the WAnT, a heart rate monitor (S810, Polar Instruments Inc., Oulu, Finland) was used to control exercise and warm-up intensity. Warm-up intensity was calculated using the re-engineered equation of Swain et al. [45] to determine the maximum heart rate percentage (%MHR) using %VO_2max_:

%MHR=0.6463×%VO2max+37.182

Where %MHR is the percentage of maximal heart rate and %VO_2max_ is the percentage of estimated maximal oxygen uptake.

A pilot study was carried out to ensure the reproducibility and sensitivity of W_peak_ and W_mean_ indices, using two measurements of 10 subjects in a single day. Both indices showed excellent intraclass correlation coefficients (ICC = 0.91–0.94), small standard error of measurements (SEM; 3.41–4.56%, <5%) and a small coefficient of variation (CV; <5%).

Similar testing procedures have been used in numerous studies involving normal weight adolescent [46], young adults [47–49], and middle-aged men [32,50–53]. The standard WAnT procedure has limitations because factors such as i) active muscle mass volume and ii) exercise bioenergetics tend to alter maximal power output (*P*max) (for review see Driss and Vanderwalle [54]. Standardized WAnT intensity has previously failed to utlize an optimal load due to heterogenous F/V profiles of individuals [44, 55–58]. According to Driss and Vanderwalle[54], the load during a standardized WAnT underestimates *P*max in normal weight and powerful adults. Moreover, the load used by researchers at the Wingate Institute was modified several times: 75g·kg^-1^ body weight[59], then 67g·kg^-1^[60], then 75g·kg^-1^[61].

### Exercise training program

Trained subjects (YT and MAT) underwent 13 weeks’CSRT as previously described [53]. Briefly, CSRT involved three consecutive sessions separated by 48 h: sprint running sessions (13 sessions), resistance training sessions (13 sessions) and sprint cycling sessions (13 sessions). Sessions were performed during the morning and lasted no longer than 70 min, inclusive of 15 min warm-up (jogging and stretching) and 15 min cool-down (jogging and stretching).

During the first training session, YT and MAT performed a sprint running session, which included three to five sets of three to five short bouts at maximum velocity. A passive recovery of 2–3 min was permitted between each set.

Forty-eight hours later, YT and MAT performed the resistance training session, which included five to six exercises targeting all major muscle groups (squat with Smith machine, machine leg extension, machine leg curl, calf raises over a step, triceps pushdown with cable machine, bicep preacher curl, and bench press. The load used during these exercises (% of one-repetition maximum [1-RM]) was progressively increased from 40 to 65% of 1-RM, and increased by 5% of 1-RM per week[62]. To produce maximal power output (i.e., velocity × load), the positive phase [63] of each exercise was performed as fast as possible [64]. The number of repetitions was maintained as 10–15 per set, and the number of sets increased from three to fourover the training period. Therefore, training volume increased progressively during the CSRT program. Rest period between sets were 3–5 min for upper body muscles[65]and at least 1 min for lower limbs to allow for tolerance to increased repetitions.To adjust loads during resistance training sessions, we determined muscle strength using a 1-RM for the six resistance exercises, before CSRT, during the sixthweek and post-CSRT. All subjects were familiarized with the test procedures. Ten-minute warm-up (stretching and cycling at 50%VO_2max_) preceded the test. After 5 min rest, subjects performed 5 repetitions at approximately 50% of the estimated 1-RM followed by another set of 3 repetitions at 70% of the estimated 1-RM. Subjects then performedone repetition of progressively heavier loads until failure. Maximum strength was determined as the maximum load that could be lifted once with proper technique.

During the third training session, subjects performed a sprint cycling session. Each series comprised three to five repetitions of 10–30 s. The 10–30 s trials were performed maximally. Subjects recovered actively (at a power output corresponding to 50%VO_2max_) for 3–5 min between each sprint.

### Blood sampling and analysis

Upon the participants’ arrival, a heparinized catheter (Insyte-W, 1.1 mm o.d. × 30 mm) was inserted into an antecubital vein, following sitting for 20 min. Blood was obtained between 08:00 and 09:00 h. to control for diurnal variation on visit two of exercise testing. Venous blood samples were drawn at four times: at rest (_0_ [after 20 min sitting on the bike]), after warm-up (_W_), immediately post-WAnT (_end_) and 10 min post-WAnT (_10_). For each sample, 10 mL of blood was collected in tubes containing ethylenediaminetetraacetic acid (EDTA) to determine concentrations of serumGH, IGF-1, and IGFBP-3. Samples were centrifuged immediately (at 3000 rpm for 15 min at 4°C), before being divided into appropriate aliquots and stored at -80°C for later analysis.

Blood sample was collected from the finger (20μL) at the third minute post-WAnT and placed in Eppendorfs for measurement of peak blood lactate concentration ([La]_peak_). Blood lactate concentration was determined using an enzymatic lactate analyzer (Microzym, Cetrix, France).

GH was assayed by chemiluminescence (Immulite, Diagnostic Products Corp., Los Angeles, CA, USA). The GH assay sensitivity limit was 0.1 ng·ml^-1^ and inter- and intra-assay CV was 5.7–10% and 4.9–8.3% respectively. IGF-1 was measured using the non-extraction IGF-1 immunoradiometric assay (IRMA) kit (Diagnostic Systems Laboratories, Webster, TX, USA). The theoretical sensitivity, or minimum detection limit, as calculated by interpolation of the mean plus twostandard deviations (SD) of 20 standard replicas 0 ng·ml^-1^ was 2 ng·ml^-1^. The inter-assay CV was 7.4% and 4.2% at concentrations of 35.5 ng·ml^-1^ and 383.9 ng·ml^-1^ respectively. Theintra-assay CV was 6.8% and 6.3% for mean concentrations of 34.03ng·ml^-1^and 373.86 ng·ml^-1^respectively. IGFBP-3 concentrations were measured by using the non-extraction IRMA kit (Diagnostic Systems Laboratories, Webster, TX, USA). The lower threshold of detection, calculated as the mean two SD of 22 standard replica 0 ng·ml^-1^ of IGFBP-3, was ~0.5 ng·ml^-1^. The intra-assay CV was 1.8% and 3.9% for mean concentrations of 82.7 ng·ml^-1^and 7.4 ng·ml^-1^ respectively. The inter-assay CV was 1.9% and 0.6% for mean concentrations of 76.9 ng·ml^-1^and 8.0 ng·ml^-1^ respectively. All biochemical assays were run in duplicate.

### Statistical analysis

Data were analyzedusing SPSS version 23.0 for Windows (SPSS, Inc. Chicago, IL, USA). Means and standard deviations (SD) were calculated after verifying normality of distributions using the Kolmogorov-Smirnov procedure. For anthropometric, physiological and physical performance, along with area under the curve (AUC), data were analyzed using a multifactorial three-way (time [P1, P2] × age [young, middle-aged] × group [trained, control]) ANOVA.

Hormone responses were analyzed using a four-factor ANOVA (time [P1, P2] × Wingate time [warm-up, immediately post-WAnT and 10 min post-WAnT] ×age [young, middle-aged] × group [trained, control]).

Total AUCs for GH, IGF-1, and IGFBP-3 were calculated to determine the total hormone exposure over the measured period. It simplified the statistical analyses by rendering the multivariate results into univariate result [66]. The AUC is determined using the linear trapezoidal method which uses linear interpolation between data points.

Greenhouse-Geisser corrections were used when the assumption of sphericity (Mauchly’s test) was violated. To help protect against type II errors, an estimate of power (ώ) and effect size (*ƞ*^2^_p_) were calculated. Bonferroni-adjusted pairwise post hoc comparisons were performed where appropriate. Pearson’s product-moment correlation coefficients were calculated to assess relationships between variables. Significance level was set *a priori* at P<0.05.

## Results

### Anthropometric data

At P1, there was a significant main effect of age for body mass (F = 4.99, P = 0.03, *ƞ*^*2*^_*p*_ = 0.13, ώ = 0.58), whereby YT and YC were significantly lighter than MAT and MAC (P<0.05). At P2, both training groups experienced a decrease in body mass from P1 (72.4±5.2 and 76.2±5.4 kg for YT, and MAT respectively [P<0.05]), whereas the control groups’ body mass was not significantly different from P1 (P>0.05). The main effect of time (from P1 to P2) was significant (F = 8.85, P<0.01, *ƞ*^2^_p_ = 0.21, ώ = 0.82) after CSRT.

At P1, there was no main effect of age for body fat percentage (11.7±1.4%, 11.3±1.8%, 12.9±1.2%, and 12.4±2.2% for YT, YC, MAT, and MAC respectively; F = 2.45, P = 0.13, *ƞ*^2^_p_ = 0.07, ώ = 0.33). At P2, both training groups experienced a decrease in body fat from P1 (10.2±0.8% and 11.2±1.4% for YT, and MAT respectively; F = 14.52, P<0.01, *ƞ*^2^_p_ = 0.30, ώ = 0.96), while control groups’ body fat percentages were not significantly different from P1 (F = 2.16, P>0.05). We also observed significant interaction between Time×Group (F = 5.82, P = 0.02, *ƞ*^2^_p_ = 0.15, ώ = 0.65).

At P1, no significant main effect of age for fat-free mass (FFM) was observed (65.2±5.5kg, 64.1±4.5kg, 62.1±5.2kg, and 60.4±3.2kg for YT, YC, MAT, and MAC respectively with F = 2.14, P = 0.15, *ƞ*^2^_p_ = 0.06, ώ = 0.30). At P2, FFM was 66.1±5.1kg, 64.6±5.7kg, 63.8±5.4kg, and 61.4±4.4 kg for YT, YC, MAT, and MAC respectively. No group experienced a significant change in FFM between P1 and P2 (P>0.05) and there was no main effect of time (F = 0.68, P = 0.41, *ƞ*^2^_p_ = 0.02, ώ = 0.13).

### Physical performance and physiological response

The results of the WAnT are displayed in Table 1. There was a significant main effect of age in W_peak_ (F = 5.98, P = 0.02, *ƞ*^2^_p_ = 0.15, ώ = 0.66). At P1, W_peak_ was significantly higher in young groups compared to middle-aged groups (P<0.05). However, this main effect of age for W_peak_ at P1 (P<0.001) was ameliorated at P2 (P>0.05).

**Table 1:** Wingate outcoumes and physiological parameters determined before (P1) and after (P2) training.

		YT (n = 10)	YC (n = 9)	MAT (n = 10)	MAC (n = 9)
**W**_**peak**_ **(W)**	**P1**	1016±126^a^^,^^d^	1000±312^e^	885±155^a^	887 ± 102
	**P2**	1050±123^c^	944 ±246^e^	997±145^b^	824 ± 113
**W**_**mean**_ **(W)**	**P1**	584 ±58	500± 93	434±86	445 ±37
	**P2**	598 ±71	473±80.6	563±67	402±80
**VO**_**2max**_ **(mL·min**^**-1**^**·kg**^**-1**^**)**	**P1**	42.2 ±6.1^a^	43.8 ±5.1	39.8 ±9.5^a^	38.5 ±3.2
	**P2**	45.5 ±5.7^c^	42.1 ±3.2	45.6 ±11.2^b^	40.1 ±3.8
**La**_**peak**_ **(mmol·l**^**-1**^**)**	**P1**	14.6 ±2.2^a^	13.9 ±3.4	13.4 ±2.7^a^	13.2 ±3.2
	**P2**	16.1 ±2.3^c^	14.3 ±3.3	15.1 ±2.6^b^	13.3 ±3.1

Data are means ±SD; maximal Power (W_peak_); mean power (W_mean_) in absolute values (W); Peak lactate concentration (La_peak_); Maximal oxygen uptake (VO_2max_); young trained (YT); young control (YC); middle-aged trained (MAT); middle-aged control (MAC); before training (P1); after training (P2).

^a^ significant differences from before and after training, ^a^: *P* < .05.

^b^ significant differences between MAT and MAC, ^b^: *P* < .05.

^c^ Significant differences between YT and YC, ^c^: *P* < .05.

^d^ significant differences between YT and MAT, ^d^: *P* < .05.

^e^ significant differences between YC and MAC, ^e^: *P* < .05.

In addition, W_peak_ increased significantly after training in both YT and MAT (P<0.05). At P2, trained groups exhibited significantly (P<0.05) higher W_peak_ compared to control groups. Significant interaction between Time×Group was registered in W_peak_ (F = 4.92, P = 0.03, *ƞ*^2^_p_ = 0.13, ώ = 0.58). No main effect of age or time (P>0.05) were observed for W_mean_.

[La]_peak_ increased significantly (P<0.05) in trained groups (YT and MAT) after CSRT (Table 1). A significant main effect of time was observed (F = 20.13, P*<*0.01, *ƞ*^2^_p_ = 0.37, *ώ =* 0.99) for [La]_peak,_ whilst there was no main effect of age (F = 1.38, P = 0.25, *ƞ*^2^_p_ = 0.04, ώ = 0.21) between groups at P2.

For VO_2max_, we observed a significant main effect of time (F = 18.35, P<0.01, *ƞ*^2^_p_ = 0.35, ώ = 0.99). Estimated VO_2max_ increased significantly after CSRT in both trained groups (P<0.001), but not in control groups (P>0.05). There was no significant main effect of age (F = 2.69, P = 0.11, *ƞ*^2^_p_ = 0.07, ώ = 0.36) in VO_2max_ between groups at P2.

### Growth hormone response

There was a significant main effect of age for GH (F = 17.47, P<0.01, *ƞ*^2^_p_ = 0.34, *ώ =* 0.98) and GH AUC (F = 18.26, P<0.01, *ƞ*^2^_p_ = 0.35, *ώ =* 1.00) (Table 2).

**Table 2:** GH concentration (ng·ml^-1^) determined before (P1) and after (P2) training.

		GH_0_	GH_w_	GH_end_	GH_10_	GH AUC
**YT (n = 10)**	**P1**	0.33±0.21^a^^,^^d^	8.65±2.26^d^	12.49±4.57^a^^,^^d^	12.51±2.55^d^	256.90±39.70^a^^,^^d^
	**P2**	0.71±0.39^c^	9.42±4.11	14.46±5.45^d^	16.74±5.36^a^^,^^c^^,^^d^	309.91±75.31
**YC (n = 9)**	**P1**	0.32±0.30^e^	8.60±1.44^e^	12.66±3.79^e^	13.31±5.22^e^	262.60±39.22^e^
	**P2**	0.45±0.03^e^	8.83±3.93^e^	13.10±2.17^e^	14.33±8.89^e^	267.20±70.04^e^
**MAT (n = 10)**	**P1**	0.24±0.31^a^	5.78±3.10 ^a^	8.15±4.23^a^	9.06±3.33^a^	187.33±36.60^a^
	**P2**	0.85±0.94^b^	8.75±2.97	10.78±1.73^b^	12.97±2.26^b^	262.91±46.72^b^
**MAC (n = 9)**	**P1**	0.13±0.01	5.99±3.96	9.02±3.30	9.64±2.52	191.71±65.62
	**P2**	0.14±0.03	8.15±2.40	10.60±1.75	11.39±1.66	208.50±17.73

Data are means ±SD; plasma GH concentration at rest (GH_0_); after warm-up (GH_w_); at the end of exercise (GH_end_); during recovery (GH_10_); GH AUC: Area under the curve (ng**·**ml^-1^ per min), young trained (YT); young control (YC); middle-aged trained (MAT); middle-aged control (MAC); before training (P1); after training (P2) and training

^a^ significant differences from before and after training, ^a^: *P* < .05.

^b^ significant differences between MAT and MAC, ^b^: *P* < .05.

^c^ Significant differences between YT and YC, ^c^: *P* < .05.

^d^ significant differences between YT and MAT, ^d^: *P* < .05.

^e^ significant differences between YC and MAC, ^e^: *P* < .05.

Significantly higher GH was observed at rest, after warm-up, immediately post- and 10 min post-WAnT in the young groups as compared to the middle-aged groups at P1 (P<0.05). This main effect of age was not present in GH_0_ or GH_w_ between MAT and YT at P2 (P>0.05).

There was a main effect of time for GH (F = 17.83, P<0.01, *ƞ*^2^_p_ = 0.34, *ώ =* 0.98) and GH AUC (F = 10.83, P<0.01, *ƞ*^2^_p_ = 0.34, *ώ =* 0.93). For YT, GH_0_, GH_end_, and GH_10_ were significantly higher at P2 compared to P1 (P<0.05). For MAT, GH_0_, GH_end_, GH_w_, and GH_10_ were significantly greater at P2 as compared to P1 (P<0.05). We observed no significant changes from P1 to P2 for GH in control groups (P>0.05).

We observed significant interaction between the Time×Group (F = 7.55, P = 0.01, *ƞ*^2^_p_ = 0.18, *ώ =* 0.76). GH_0_ and GH_10_ (both P<0.01) were significantly higher in YT compared to YC at P2. GH_0_, GH_end_, and GH_10_ were significantly higher in MAT compared to MAC at P2 (P<0.05).

### IGF-1 response

A significant main effect of Wingate-time (F = 203.19, P<0.01, *ƞ*^2^_p_ = 0.86, *ώ =* 1.00) was observed in all groups (Table 3).

**Table 3:** IGF-1 concentrations (ng·ml^-1^) determined before (P1) and after (P2) training.

		IGF-1_0_	IGF-1_w_	IGF-1_end_	IGF-1_10_	IGF-1 AUC
**YT (n = 10)**	**P1**	327.60±137.54^d^	383.80±169.02^a^^,d^	417.91±172.67^a^^,d^	678.81±117.33^d^	13767.70±4425.0^a^^,^^d^
	**P2**	340.90±130.97^d^	436.79±176.66^d^	556.63±136.98^c^	749.87±113.10^c^^,^^d^	15886.71±4034.13
**YC (n = 9)**	**P1**	340.22±154.45^e^	323.65±135.19^e^	433.73±113.92^e^	465.40±58.12^e^	13846.01±2541.70^e^
	**P2**	361.78±139.05^e^	353.18±146.30^e^	443.82±100.05^e^	528.12±118.73^e^	12994.21±3763.7^e^
**MAT (n = 10)**	**P1**	157.10±54.0 ^a^	223.78±53.25^a^	292.55±61.62 ^a^	300.44±58.11	8379.40±1172.31^a^
	**P2**	191.00±66.42	268.11±70.56	458.39±71.78 ^b^	392.03±84.64	10403.32±1032.53^b^
**MAC (n = 9)**	**P1**	150.89±87.83	257.58±46.14	358.89±75.70	401.58±78.95	9077.52±1363.22
	**P2**	153.88±59.25	210.43±44.97	238.29±38.88	331.73±40.52	7951.21±1192.63

Data are means ±SD; plasma IGF-1 concentration at rest (IGF-1_0_); after warm-up (IGF-1_w_); at the end of exercise (IGF-1_end_); during recovery (IGF-1_10_), IGF-1 AUC: Area under the curve (ng**·**ml^-1^ per min), young trained (YT); young control (YC); middle-aged trained (MAT); middle-aged control (MAC); before training (P1); after training (P2).

^a^ significant differences from before and after training, ^a^: *P* < .05.

^b^ significant differences between MAT and MAC, ^b^: *P* < .05.

^c^ Significant differences between YT and YC, ^c^: *P* < .05.

^d^ significant differences between YT and MAT, ^d^: *P* < .05.

^e^ significant differences between YC and MAC, ^e^: *P* < .05.

At P1 and P2, IGF-1 increased throughout the progression of the WAnT in all groups (P<0.05), i.e., from IGF-1_0_ to IGF-1_10_.In addition, significant interaction between Wingate-Time×Age (F = 16.25, P<0.001, *ƞ*^2^_p_ = 0.32, *ώ* = 1.00) Wingate-Time×Group (F = 3.57, P = 0.02, *ƞ*^2^_p_ = 0.10, *ώ* = 0.78), Wingate-Time×Time (F = 12.82, P<0.001, *ƞ*^2^_p_ = 0.27, *ώ* = 1.00), Wingate-Time×Time×Group (F = 5.00, P<0.001, *ƞ*^2^_p_ = 0.13, *ώ* = 0.91), and Wingate-Time ×Time×Group×Age (F = 3.71, P = 0.01, *ƞ*^2^_p_ = 0.10, *ώ* = 0.79) were observed in IGF-1.

We observed a significant main effect of age for IGF-1 (F = 42.08, P<0.01, *ƞ*^2^_p_ = 0.55, *ώ =* 1.00) and IGF-1 AUC (F = 41.02, P<0.01, *ƞ*^2^_p_ = 0.50, *ώ =* 1.00). IGF-1 was significantly higher at all phases of the WAnT in young as compared to middle-aged groups at P1 (P<0.05). However, this age effect was not present at P2 (P>0.05 between YT and MAT), whilst, for the control groups, the main effect of age remained statistically significant at P2 (P<0.01). A main effect of time was observed in IGF-1 AUC (F = 30.42, P = 0.01, *ƞ*^2^_p_ = 0.17, *ώ =* 1.00). Significantly greater IGF-1_w_ and IGF-1_end_ in YT, and IGF-1_10_, IGF-1_w_, and IGF-1_end_ was observed at P2 compared to P1 in MAT (P<0.05). When compared to control groups, significantly greater IGF-1_end_ was observed in MAT compared to MAC at P2 (P<0.05). This last result is confirmed by a significant interaction between Time×Group (F = 60.90, p = 0.00, *ƞ*^2^_p_ = 0.64, *ώ =* 1.00).

### IGFBP-3 response

There was a significant main effect of Wingate-Time (F = 13.50, P<0.01, *ƞ*^2^_p_ = 0.28, *ώ =* 1.00). At P1 and P2, IGFBP-3 increased throughout the progression of the WAnT in all groups (P<0.05), i.e., from IGFBP-3_0_ to IGFBP-3_10_ (Table 4).

**Table 4:** IGFBP-3 concentrations (ng·ml^-1^) determined before (P1) and after (P2) training.

		IGFBP-3_0_	IGFBP-3_w_	IGFBP-3_end_	IGFBP-3_10_	IGFBP-3AUC
**YT (n = 10)**	**P1**	4072±389^d^	4019±422	4220±333^d^	4100±449^d^	132162±12088
	**P2**	4163±328 ^c^	4211±405	4330±350 ^c^	4349±398 ^c^^, d^	13174±8902^c^
**YC (n = 9)**	**P1**	3749±403^e^	3886±358^e^	4012±384^e^	3677±373	123202±12087
	**P2**	3788±349^e^	3826±305^e^	4022±335^e^	3632±348	122455±5192^e^
**MAT (n = 10)**	**P1**	3060±389 ^a^	3743±290 ^a^	3596±295	3759±372	112767±9871
	**P2**	4130±416	4010±294	3951±238^b^	3936±391^b^	124857±6672^b^
**MAC (n = 9)**	**P1**	3373±403	3613±332	3789±366	3775±383	111005±7022
	**P2**	3332±379	3648±328	3763±357	3731±375	111860±9854

Data are means ±SD; plasma IGFBP-3 concentration at rest (IGFBP-3_0_); after warm-up (IGFBP-3_w_); at the end of exercise (IGFBP-3_end_); during recovery (IGFBP-3_10_), young trained (YT); IGFBP-3 AUC: Area under the curve (ng**·**ml^-1^ per min), young control (YC); middle-aged trained (MAT); middle-aged control (MAC); before training (P1); after training (P2)

^a^ significant differences from before and after training, ^a^: *P* < .05.

^b^ significant differences between MAT and MAC, ^b^: *P* < .05.

^c^ Significant differences between YT and YC, ^c^: *P* < .05.

^d^ significant differences between YT and MAT, ^d^: *P* < .05.

^e^ significant differences between YC and MAC, ^e^: *P* < .05.

There was a significant main effect of age for IGFBP-3 (F = 25.57, P<0.01, *ƞ*^2^_p_ = 0.43, *ώ =* 1.00). Significantly higher basal IGFBP-3 was observed for YT and YC compared to MAT and MAC at P1 (P = 0.001 and P = 0.002 respectively). However, this main effect of age was not observed at P2 (i.e., between YT and MAT at P2; P>0.05).

Significant increases in IGFBP-3 were observed in MAT at rest (P = 0.001) and during warm-up (P = 0.003) at P2 compared to P1. When compared to control groups, IGFBP-3_0_, IGFBP-3_end_, and IGFBP-3_10_ were significantly higher in trained groups compared to control groups at P2 (P<0.05). In fact, a main effect of group was also observed (F = 14.12, P<0.01, *ƞ*^2^_p_ = 0.29, *ώ =* 0.95).

### IGF-1/IGFBP-3 ratio

**Fig 1:**
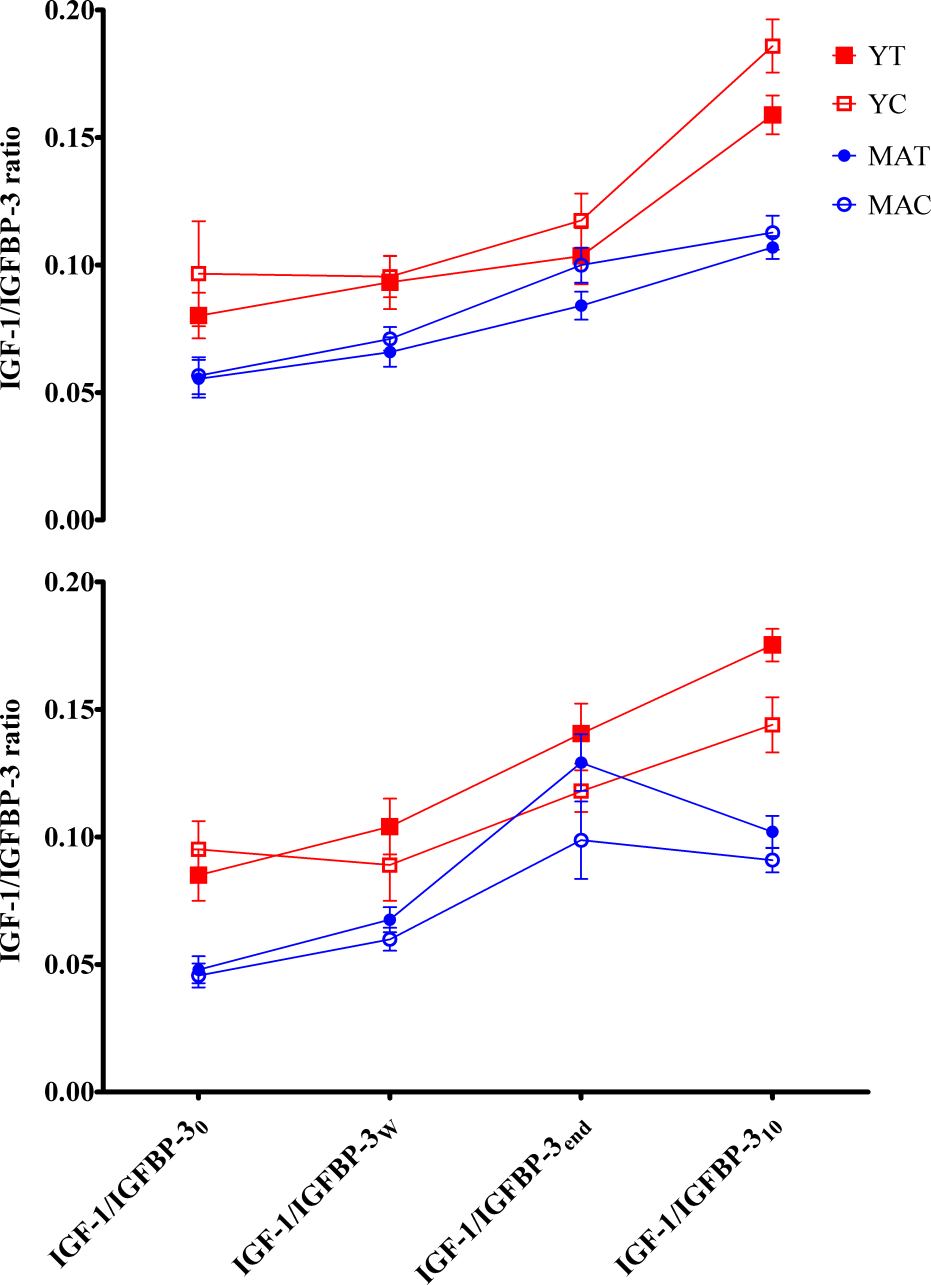
[IGF-1]/[GFBP-3] ratio changes determined before (P1; above) and after (P2; below) training. Plasma [IGF-1]/[GFBP-3] ratio at rest (_0_), after warm-up (_w_), at the end of exercise (_end_), and during recovery (_10_) in young trained (YT), young control (YC), middle-aged trained (MAT), and middle-aged control (MAC) groups. Data are presented as mean ± standard error for clarity.

The IGF-1/IGFBP-3 ratio was affected by Wingate-time (F = 130.27, P<0.01, *ƞ*^2^_p_ = 0.79, *ώ =* 1.00). Post-hoc Bonferroni adjustments indicated the ratio increased significantly (P<0.05) from rest to the end of exercise in all groups at P1 and P2. i.e. from IGF-1/IGFBP-3_0_ to IGF-1/IGFBP-3_10_ (Fig 1).

A main effect of age (F = 25.57, P<0.01, *ƞ*^2^_p_ = 0.43, *ώ =* 1.00) was observed for IGF-1/IGFBP-3 at P1 and was significantly higher at all phases of the WAnT in young compared to middle-aged groups (P<0.05). However, this age effect was not present following CSRT (P>0.05 between YT and MAT), whilst, for control groups, the main effect of age remained statistically significant at P2 (P<0.01). Significant interactions were observed between Age×Group (F = 14.29, P<0.01, *ƞ*^2^_p_ = 0.30, *ώ =* 1.00). CSRT induced significant increases in IGF-1/IGFBP-3_w_ and IGF-1/IGFBP-3_end_ in YT, and increases in IGF-1/IGFBP-3_10_, IGF-1/IGFBP-3_w_ and IGF-1/IGFBP-3_end_ in MAT (P<0.05). When compared to control groups, significantly higher IGF-1/IGFBP-3_end_ were observed in MAT as compared to MAC at P2 (P<0.05) with significant interactions observed between Time×Group (F = 32.82, P<0.01, *ƞ*^2^_p_ = 0.49 and *ώ =* 1.00).

### Correlative analysis

Before training, a significant positive correlation existed between GH_0_ and W_peak_ for YT (r = 0.563, P = 0.003). GH_end_ was also positively correlated with W_peak_ at P1 (r = 0.792, P = 0.001) and P2 (r = 0.710, P<0.001) in MAT, but only at P2 in YT (r = 0.268, P = 0.002).

## Discussion

The primary finding of the present investigation is that 13 weeks’ CSRT induced a significant increase in W_peak_ in both YT and MAT which ameliorated the effect of age between these groups. Increased sprint performance was associated with increased GH at rest, in response to the WAnT, and during recovery in YT and MAT, whilst IGF-1 and IGFBP-3 increased at rest in MAT only. Prior to CSRT, an effect of age was observed in GH, IGF-1, IGFBP-3, and IGF-1/IGFBP-3 basally and in response to exercise. However, CSRT ameliorated this age effect as both training groups (MAT and YT) had comparable basal GH and GH AUC, IGF-1_end_ and IGF-1 AUC, and IGF-1/IGFBP-3.

The results of the AUC analysis for GH and IGF-1 suggest CSRT produce prolonged elevations in these hormones in response to the WAnT. Moreover, the increased IGF-1/IGFBP-3 ratio in response to exercise in trained groups suggests that there may be more unbound IGF-1 available for hormone-receptor interactions.

GH increases are associated with improvements in lean body mass and reduced fat mass [67] which was supported by the present investigation. In the current study, trained groups exhibited a trend for greater FFM at P2 (~0.9–1.7kg increases) and significantly less fat mass which may increased oxidative capacity as more active tissue would permit a greater oxygen uptake [68].Our observation of increased estimated VO_2max_ of ~16% following CSRT is in line with previous investigators who report increased cardiorespiratory fitness following high intensity training [69, 70]. In their recent review, Sloth et al. [71] suggested short term sprint interval training performed alone induces increases of 4.2–13.4% VO_2max_ in young adult males. Therefore, the combination of sprint interval training with strength exercise appears a pragmatic approach to improve aerobic capacity in young and middle-aged subjects. These adaptations may be explained by increased enzymatic activity and mitochondrial mass [71–73]. However, a mechanistic explanation is outside the scope of this investigation.

As increased aerobic capacity is associated with increased blood lactate during intense efforts[54, 74], the higher blood lactate observed in trained groups may be due to increased ATP hydrolysis leading to greater lactate efflux in the plasma compartment as supported previously [53, 75].

CSRT produced a significant increase in W_peak_ in trained groups, which supports several studies that report anaerobic training leading to enhanced power production in young [76] and older [77, 78] subjects. Interestingly, we found a positive correlation between serum growth factors and physical performance alterations, providing further associative evidence linking growth factors to improved muscle power [79].

In the present study, training-induced increases in basal GH occurred between trained groups, and ameliorated the age effect at P2. Such findings are in accordance with recent investigations demonstrating that intense training increases GH in males [80, 81]. However, other studies have not observed perturbations to basal GH after sprint [34, 82, 83] or resistance [83] training performed alone.The relatively short half-life of GH and the “snapshot” nature of basal hormone sampling may explain discrepancies in resultsas GH during rest loses half of its physiologic activity in10-30 minutes following secretion [84]. This brief period of hormonal activity depends on several factors such as diet, sleep duration, blood sampling (stress response from the vascular puncture) and time of the day [20, 84] which contributes to fluctuations in basal GH. The most reproducible pulse of GH secretion however, occurs in response to intense exercise, when rate of secretion exceeds rate of degradation [85]. For example, Nevill et al. [34]reported GH to be tenfold higher than basal levels in sprinters following exercise and were greater than those measured in endurance athletes. GH increases may also be exercise volume dependent as 30s all-out sprints resulted in greater GH response than 6 s sprints and remained high for >90 minutes compared to 60 min following 6 s sprint [86]. An alternative explanation for discrepancies with previous investigations is the utilisation of concurrent trainingin the present study, rather than only resistatnce or only sprint training previously investigated.

GH anabolic actions are mostly mediated through IGF-1 and include regulation of whole body protein synthesis and breakdown[20]. Interestingly, the increased GH AUC following CSRT was associated with increase in IGF-1 AUC in YT and MAT suggesting that CSRT is a potent stimulus of the GH/IGF-1 axis. Data from the present investigation are in agreement with some [87] but not all [88] previous investigations in reporting increased basal IGF-1 following exercise training. For example, Cooper, Taaffe (76] reported comparable IGF-1 in masters runners and minimally exercising men aged 60–70 yrs (129±10 ng·ml^-1^and 124±11 ng·ml^-1^, respectively). Differences in exercise modality and participant characteristics may explain discrepancies in findings, as the present investigation studied the influence of CSRT in young and middle-aged, rather than older, men as in the aforementioned investigation. Whilst increased systemic IGF-1 is generally considered to confer an anabolic advantage, a recent investigation [89] reported decreased systemic IGF-1 following 12 weeks’ resistance exercise in older adults (74±6yrs), despite increased lean mass. These authors concluded that, during periods of active muscle building, IGF-1 is redistributed from circulation into tissue. Moreover, increased IGF-1 was observed without significant alteration in IGFBP-3 AUC in MAT following CSRT. IGFBP-3 is known to enhance IGF-1 retention in the circulation and block its access to the cognate receptor (IGF-1R)[15, 16]. The effect of intense training on IGFBP3 in elderly is still conflicting[90], and further investigation in large population is needed to better understand its variability over time in response to intense training.

There is sufficient evidence that anabolism in the elderly are intimately tied to alterations in GH/IGF-1 axis and declines in total and free IGF-1 response [91]. Nevertheless, CSRT appears to reduce this age-related effect on IGF-1/IGFBP-3 (a surrogate for IGF-1 bioavailability) between trained groups at rest, at the end of WAnT, and during recovery with important increases at all phases following the WAnT. IGF-1 bioavailability appears a strong predictor on the effectiveness of the IGF-1 action [92] and its increase confirms improvements of GH/IGF-1 axis in young and middle-aged trained men with training.

## Conclusion

In conclusion, 13 weeks’ CSRT improved peak power output and body composition in young and middle-aged military men. This training intervention increases circulating GH in trained groups at rest and in response to exercise. Training-induced increases in basal GH occurred with amelioration of the age effect between trained groups. GH improvements in middle-aged men were associated with increased IGF-1 levels following CSRT. These were associated with increased IGF-1 bioavailability index in young and middle-aged trained groups following training. As such, CSRT appears to counteract the aging effect of somatotropic hormones.
